# Migraine and the Risk of Dementia in the General Population

**DOI:** 10.1002/alz.71386

**Published:** 2026-04-23

**Authors:** Cevdet Acarsoy, Daniel Bos, Sanne S. Mooldijk, M. Arfan Ikram, M. Kamran Ikram

**Affiliations:** ^1^ Department of Epidemiology Erasmus MC University Medical Center Rotterdam Rotterdam The Netherlands; ^2^ Department of Radiology & Nuclear Medicine Erasmus MC University Medical Center Rotterdam Rotterdam The Netherlands; ^3^ Department of Neurology Erasmus MC University Medical Center Rotterdam Rotterdam The Netherlands

**Keywords:** Alzheimer's disease, APOEε4, dementia, hazard ratios, longitudinal follow‐up, migraine, neurodegeneration, prospective cohort study, risk factors, Rotterdam study

## Abstract

**INTRODUCTION:**

Migraine and dementia are prevalent disorders with high societal impact; however, research regarding their association remains inconsistent.

**METHODS:**

We analyzed 6888 participants from the prospective Rotterdam Study. Migraine status was assessed (2006–2011) via validated questionnaire. Participants were followed for a median of 9.4 years until January 2020. Dementia diagnoses were confirmed through in‐person screenings and medical record linkages, including neuroimaging.

**RESULTS:**

At baseline, 1041 participants (15.1%) had migraine. Over 62,180 person‐years, 491 participants developed dementia (379 [77.2%] with Alzheimer's). Cox models revealed that migraine was associated with a lower risk of dementia (hazard ratio [HR] 0.70, 95% confidence interval [CI]: 0.51–0.95) and Alzheimer's disease (HR 0.58, 95% CI: 0.40–0.85).

**DISCUSSION:**

This study suggests that migraine is associated with a reduced risk of dementia, including Alzheimer's disease. Whether this reflects biological neuroprotection or methodological factors is currently unclear, necessitating further investigation.

## Highlights

- Migraine is not associated with an increased risk of dementia in a population‐based study.
- Findings challenge previous hypotheses linking migraine to cognitive decline mechanisms.
- Possible protective factors of migraine against dementia warrant further investigation.
- Results highlight the need for longitudinal studies incorporating neuroimaging and biomarkers.

## 1 BACKGROUND

It has been suggested that migraine in early life may be related to an increased risk of dementia, including Alzheimer's disease (AD).^1^ Though not fully understood, proposed mechanisms linking these two conditions include neurovascular disease, psychosocial factors (e.g., depression and stress), and structural changes in overlapping brain regions involved in chronic pain and memory functions.^2^ Several meta‐analyses^1^, ^3^, ^4^, ^5^ suggest that individuals with migraine are at an increased risk of dementia. However, the true nature of this association remains unclear, partly due to methodological limitations. Most of the studies included in these meta‐analyses have either cross‐sectional or retrospective design.

One such recent meta‐analysis, published in 2024, focused on primary headache, including migraine. Of the 23 included studies, 15 were cross‐sectional or retrospective in nature, while only eight were prospective cohort studies.^3^ In the overall meta‐analysis, combining 18 studies, there was a suggestion of a positive associations of primary headache (odds ratio [OR] 1.15, 95% confidence interval [CI]: 1.03‐1.28) and dementia, with similar findings for migraine (OR 1.26, 95% CI: 1.13‐1.40). However, when prospective studies were analyzed separately, an inverse relationship was observed. Primary headache (OR 0.82, 95% CI: 0.74‐0.92) and migraine (OR 0.88, 95% CI: 0.73‐1.05) seemed to decrease the risk of dementia. A critical assessment of these findings from previous meta‐analyses suggests that the link between migraine and dementia is not straightforward.

**RESEARCH IN CONTEXT**

1. **Systematic review**: The authors searched PubMed for epidemiological cohort studies and recent meta‐analyses. Findings are inconsistent; while retrospective data often suggest an increased risk, prospective cohort studies demonstrate an inverse or non‐significant relationship. This is addressed by utilizing a large, population‐based prospective cohort.
2. **Interpretation**: Study findings show that migraine is associated with a 30% reduced risk of all‐cause dementia and a 42% reduced risk of Alzheimer's disease. These results challenge the traditional risk‐factor hypothesis and suggest a potential neuroprotective role. Besides methodological considerations, the results may support the idea that migraine attacks may serve as a homeostatic response to oxidative stress, potentially slowing neurodegenerative processes.
3. **Future directions**: Research should investigate the biological mechanisms underlying this effect, specifically oxidative stress regulation. Also, longitudinal studies are needed to determine how migraine severity, frequency, subtypes (e.g., with aura), and pharmacological treatments independently influence long‐term dementia risk.

Findings from the existing literature cannot be given a simple causal interpretation because of several well‐known methodological limitations: Firstly, cross‐sectional and retrospective studies are susceptible to recall bias.^3^ Secondly, the association between migraine and dementia reported in the literature may reflect selection bias, since individuals are usually recruited from hospitals and they may include more severe cases of migraine compared to the general population. Finally, as migraine typically occurs in younger age groups compared to dementia, understanding the temporal relationship between migraine and dementia requires a long follow‐up period that encompasses the prodromal phase of dementia, which was not the case in several studies.^5^ Given the limitations in existing literature, we determined the association between migraine and the risk of dementia in a population‐based prospective cohort study.

## 2 METHODS

### 2.1 Study setting and population

This study was embedded in the Rotterdam Study, a prospective population‐based cohort study among middle‐aged and older community residents of Ommoord, Rotterdam, the Netherlands.^6^ The original cohort of the study started in 1990, followed by an expansion cohort in 2000, and both cohorts included participants ≥55 years old. In 2006, the Rotterdam Study expanded with another cohort that included participants ≥45 years old. Participants were followed for incident dementia, starting from migraine interview until incident dementia, death, last date when they were known to be dementia‐free, or January 1, 2020 (end of follow‐up), whichever came first.

### 2.2 Assessment of migraine

Migraine was assessed during a structured interview with a questionnaire based on the International Classification of Headache Disorders, second edition (ICHD‐2) criteria.^7^ Items were modified from the questionnaire validated for use in the Genetic Epidemiology of Migraine study.^8^ The questionnaire had a sensitivity of 0.93 and a specificity of 0.36. The lifetime history of migraine was assessed using the following diagnostic criteria: minimum five attacks of severe pain lasting 4‐72 hours (when untreated) with at least two of the four characteristics (unilateral location, pulsating quality, severe pain intensity, and worsening by or causing avoidance of routine physical activity), and any of the two characteristics (nausea; photophobia, and phonophobia), with these symptoms not being attributed to another disorder. Migraine with aura was defined as reporting headache attacks with visual, sensory, or language‐related transient focal neurological symptoms (i.e., aura) that lasted between 5 and 60 minutes. All participants who met the criteria for lifetime history of migraine or active migraine (<1 year since the last attack) were categorized as persons with migraine. Participants who meet all but one criterion for migraine without aura are categorized as probable migraine and considered participants without migraine in the analysis.

As described previously,^9^ our questionnaire was slightly modified from ICHD‐2 criteria in two ways: First, the first question served as a screening tool: only participants who had reported a history of severely (instead of moderately) painful headaches in a screening question received the rest of the questions. Second, for migraine with aura, participants were required to meet the criteria for migraine without aura (i.e., at least five attacks), after which we assessed for aura symptoms (visual, sensory, or language‐related). This design was used to ensure that only individuals with a clear history of migraine without aura were included before assessing for aura, which differs from the standard ICHD‐2 criteria where only two attacks are required for migraine with aura.

### 2.3 Assessment of dementia

Participants were screened for dementia at baseline and subsequent center visits via the Mini‐Mental State Examination (MMSE; cutoff <26) and Geriatric Mental Schedule (GMS) organic level (cutoff > 0).^10^ Screened positive participants were further tested using the Cambridge examination for mental disorders of the elderly (CAMDEX).^10^ In addition, dementia diagnoses were supplemented by linkage with general practitioners’ medical records, including reports from neurologists and geriatricians, to ensure continuous surveillance of participants, even those who no longer attended research visits.

Alzheimer's disease diagnosis was made based on clinical symptoms, disease course, and available diagnostic testing, including MRI scans. The final diagnosis was determined through consensus meetings led by experienced neurologists, using DSM‐III‐R (Diagnostic and Statistical Manual of Mental Disorders ‐ Third Revision) criteria for dementia and NINCDS‐ADRDA (National Institute of Neurological and Communicative Disorders and Stroke‐Alzheimer's Disease and Related Disorders Association for AD) criteria for AD.

### 2.4 Other measurements

Detailed information on variables was collected via interview, physical examination, and laboratory measurements. Cardiometabolic variables were body mass index (weight in kg divided by height in m^2^), hypercholesterolemia (total cholesterol ≥6.2 mmol/L or lipid‐lowering medication use), hypertension (blood pressure systolic ≥140 mm Hg, diastolic ≥90 mm Hg or antihypertensive medication use), and diabetes mellitus (fasting plasma glucose level ≥ 7.0 mmol/L or use of antidiabetic medication or of insulin). Moreover, the following variables were measured via questionnaires: marital status (single, married/cohabiting, widowed, divorced/separated), educational level (primary, lower/intermediate education, intermediate vocational or higher general education, and higher vocational education or university), smoking (never, former, and current smoker), history of coronary heart disease (myocardial infarction or revascularization), alcohol use (grams/day), depressive symptoms (Center for Epidemiologic Studies Depression Scale score),^11^ and diagnosis of at least one anxiety disorder (Munich Composite International Diagnostic Interview).^12^ Finally, apolipoprotein E (*APOE) ‐ε4* carriership was assessed by polymerase chain reaction in the first cohort and bi‐allelic TaqMan assay in later cohorts on coded DNA samples without the knowledge of dementia diagnosis.^13^ It was categorized as no ε4 allele, carrier of one ε4 allele or carrier of two ε4 alleles.

### 2.5 Population for analysis

A total of 7266 participants were interviewed to determine their migraine status. Lifetime migraine prevalence was assessed between 2006 and 2011 in a home interview or over the telephone (*n* = 634, due to logistic reasons). The overall response rate for the migraine interview was 91.1% (first cohort: 71.7%; second cohort: 96.8%; third cohort: 98.9%). Participants who did not provide informed consent (*n* = 66), with insufficient information on dementia screening (*n* = 258) or with prevalent dementia (*n* = 54) were excluded. This left us with a total sample size of 6888 participants. Dementia follow‐up extended until January 1, 2020, after which the dataset was finalized for analysis in 2022. The available follow‐up time was consistent with the study's data collection schedule, limiting further data analysis beyond this point.

### 2.6 Statistical analyses

To assess the effect of migraine on dementia risk, we applied the following strategy. First, we constructed two Cox regression models. Model 1 was adjusted for age and sex. Model 2 was further adjusted for educational level, smoking status, body mass index and *APOE‐ε4* carriership. Second, we repeated these analyses with AD as the outcome. These models were also stratified by sex. Third, we also analyzed how migraine related to the risk of mortality to assess the possible effect of competing risk in our associations. Competing risks, such as death occurring before the onset of new cases of dementia, are treated as independent events, which are often overlooked in conventional methods of survival analysis. Therefore, the actual observation of our event of interest may be obstructed by the presence of such competing risks. This could potentially skew the results of the association we are investigating.^14^ To incorporate competing risks in our estimations of the cumulative incidence rates for both dementia and all‐cause mortality, sub‐distribution hazard models were used for the unadjusted survival analyses.^15^ Fourth, we stratified the results by *APOE‐ε4* carriership, hypertension, diabetes mellitus, smoking status, age group, and study cohort to determine whether there was an effect modification on the association between migraine and dementia. Finally, we conducted a subgroup analysis where we subset the data to include only migraine patients and examined the relationship between migraine medication use and dementia risk, adjusting for the same variables in model 2.

Based on the previous research, we considered body mass index,^16^, ^17^ educational level (as a proxy measure for socioeconomic status),^16^, ^18^ smoking^16^, ^18^ and *APOE‐ε4* carriership^16^, ^19^ as common causes of migraine and dementia. Notably, other variables such as hypertension,^16^, ^20^ hypercholesterolemia,^21^, ^22^ alcohol use,^16^, ^23^ diabetes mellitus,^16^, ^24^ coronary heart disease history,^16^, ^25^ depression,^16^, ^26^ marital status,^27^, ^28^ and anxiety^29^, ^30^ could be considered as mediators in the migraine–dementia relationship. Hence, we excluded these variables from the adjustment set to avoid overadjustment bias in our main analyses. However, to test whether their inclusion impacts the model estimates, we decided to conduct additional sensitivity analyses, which are included in the supplementary materials.

Proportionality assumptions for Cox Models were checked visually via log‐minus‐log plots and Schoenfeld residuals test. IBM SPSS version 25^31^ and R version 4.0.5^32^ for Windows were used for the analyses. Cox models were fitted using “survival” R package (version 3.2‐11).^33^ Results were visualized using the “ggplot2” R package version 3.3.3.^34^ Missing values among covariables were imputed using multiple imputations by chained equation based on all variables but migraine and dementia, with five imputations and 30 iterations using “mice” R package (version 3.13.0).^35^

## 3 RESULTS

The baseline characteristics of the study population are shown in Table 1. The mean age of the population was 66.1 (±11.3) years, 57.3% were female, and 1041 (15.1%) had migraine. Of the participants with migraine, 397 (38.1%) had active migraine and 213 (20.4%) had migraine with aura. During 62,180 person‐years of follow‐up, 1895 participants died (incidence rate [IR] 30.5/1000 person‐years at risk, 95% CI: 29.1‐31.9) and 491 participants were diagnosed with dementia (IR 7.9/1000 person‐years at risk, CI: 7.2‐8.6). Among those 379 (77.2%) had Alzheimer's disease.

**TABLE 1:** Baseline characteristics categorized by migraine at baseline.

		Migraine		
Parameter	Total (*N* = 6888)	No (*N* = 5847)	Yes (*N* = 1041)	*p*‐value
**Study cohort**				<0.001
First	1509 (22%)	1325 (23%)	184 (18%)	
Second	1802 (26%)	1530 (26%)	272 (26%)	
Third	3577 (52%)	2992 (51%)	585 (56%)	
**Age (years)**	66.1 (± 11.3)	66.3 (± 11.4)	64.8 (± 10.8)	<0.001
**Female**	3947 (57%)	3101 (53%)	846 (81%)	<0.001
**Migraine subgroups**				
Active migraine	397 (6%)	*NA*	397 (38%)	
Presence of aura	213 (3%)	*NA*	213 (20%)	
Migraine medication use*	406 (6%)	*NA*	406 (39%)	
**Educational level**				0.003
Primary	629 (9%)	535 (9%)	94 (9%)	
Intermediate	2758 (40%)	2291 (39%)	467 (45%)	
Higher	2035 (30%)	1770 (30%)	265 (25%)	
University	1466 (21%)	1251 (21%)	215 (21%)	
**CHD history**	553 (8%)	512 (9%)	41 (4%)	<0.001
**Hypercholesterolemia**	3535 (51%)	2988 (51%)	547 (53%)	0.41
**Hypertension**	4716 (68%)	4021 (69%)	695 (67%)	0.212
**Body mass index** (**kg/m** ^2^)	28 (± 4.4)	28 (± 4.4)	28 (± 4.7)	0.418
**Diabetes mellitus**	964 (14%)	851 (15%)	113 (11%)	0.002
**Alcohol consumption (g/day)**	7.7 (± 8.8)	8.1 (± 9.1)	5.7 (± 6.6)	<0.001
**Smoking status**				<0.001
Past	3452 (50%)	2933 (50%)	519 (50%)	
Current	1151 (17%)	1014 (17%)	137 (13%)	
**Marital status**				0.031
Single	284 (4%)	235 (4%)	49 (5%)	
Married/cohabiting	4921 (71%)	4217 (72%)	704 (68%)	
Widowed	1012 (15%)	841 (14%)	171 (16%)	
Divorced/separated	671 (10%)	554 (9%)	117 (11%)	
**Depressive symptoms (CES‐D score)**	5.7 (± 7.3)	5.4 (± 7.0)	7.5 (± 8.4)	<0.001
**≥ 1 Anxiety disorder (M‐CIDI)**	480 (7%)	367 (6%)	113 (11%)	<0.001
***APOE‐ε4*** **carriership**				0.158
No *ε4* allele	4959 (72%)	4221 (72%)	738 (71%)	
1 *ε4* allele	1774 (26%)	1488 (25%)	286 (27%)	
2 *ε4* allele	155 (2%)	138 (2%)	17 (2%)	

*Note*: Values are presented either as mean ± SD or as number (%). Number of missing values before imputation: 2 (0.03%) for smoking, 4 (0.06%) for marital status, 13 (0.19%) for alcohol consumption, 40 (0.58%) for depressive symptoms, 61 (0.89%) for educational level, 85 (1.23%) for diabetes mellitus, 143 (2.08%) for hypertension, 158 (2.29%) for anxiety disorder, 292 (4.24%) for hypercholesterolemia, 262 (3.8%) for body mass index, 306 (4.44%) for coronary heart disease history, 579 (8.41%) for APOE‐ε4 carriership, and 808 (11.73%) for migraine medication.

Abbreviations: *APOE‐ε4*, apolipoprotein E*‐ε4*; CES‐D, Center for Epidemiologic Studies Depression Scale; CHD, coronary heart disease (myocardial infarction or revascularization); M‐CIDI, Munich‐Composite International Diagnostic Interview;

* Self‐reported prescription of triptans or ergotamines.

**FIGURE 1:**
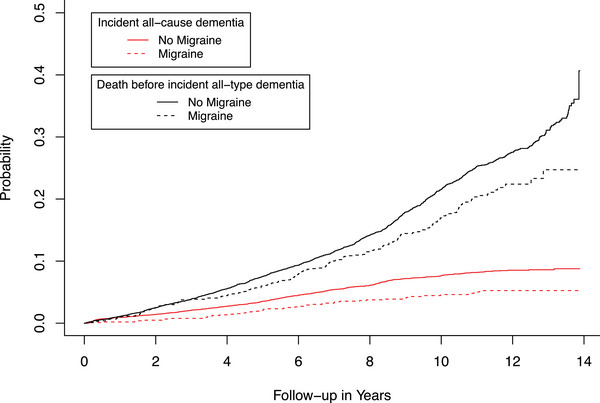
Cumulative incidence curves for all‐cause mortality and incident dementia according to migraine status.

**FIGURE 2:**
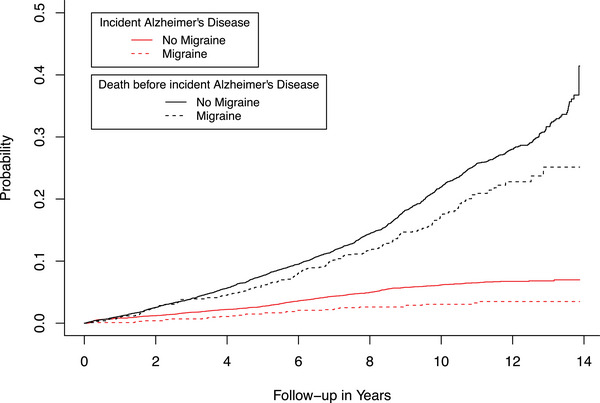
Cumulative incidence curves for all‐cause mortality and incident Alzheimer's disease according to migraine status.

In multivariable adjusted Cox regression models, we found that participants with migraine had a lower risk of dementia (hazard ratio (HR) of 0.70 (CI: 0.51‐0.95) (Table 2). Additionally, for the association between migraine and the risk of AD, we found a HR of 0.58 (CI: 0.40‐0.85; Table 2). Furthermore, a competing risk model was implemented to evaluate the impact of mortality as a competing risk during the follow‐up period. Participants with migraine had lower cumulative incidence of all‐cause mortality than participants without migraine. Moreover, participants with migraine exhibited lower cumulative incidence of all‐cause dementia (Figure 1) and Alzheimer's disease (Figure 2). Sex‐specific analysis did not suggest an effect modification by sex. Effect estimates for the risk of dementia were showing the same direction for both sexes, although only the results in females reached statistical significance (males, HR 0.71, CI: 0.32‐1.62; females, HR 0.69, CI: 0.50‐0.97; Table 2).

**TABLE 2:** Cox regression analyses for the association of migraine and the risk of dementia and Alzheimer's disease.

	Hazard ratio (95% confidence interval)			
	Cases/N	Dementia	Cases/N	Alzheimer's disease
Migraine				
Model 1^a^	491/6888	0.71 (0.52–0.96)	379/6888	0.60 (0.41–0.86)
Model 2^b^	491/6888	0.70 (0.51–0.95)	379/6888	0.58 (0.40–0.85)
Sex‐specific analysis^c^				
Males	183/2941	0.71 (0.32–1.62)	135/2941	0.84 (0.34–2.08)
Females	308/3947	**0.69 (0.50–0.97)**	244/3947	**0.55 (0.36–0.83)**

*Note*: Estimates written in bold denote statistical significance at the alpha 0.05 level.

Abbreviation: *APOE‐ε4*, apolipoprotein E‐ε4.

^a^ Model 1: adjusted for age and sex.

^b^ Model 2: Model 1 + educational level, smoking status, body mass index and *APOE‐ε4* carriership adjusted.

^c^ Models are adjusted for age, educational level, smoking status, body mass index and *APOE‐ε4* carriership.

To assess the robustness of our findings, we conducted sensitivity analyses where we explored potential effect modifiers and tested whether the inclusion of previously excluded covariates altered the results. Effect modification analyses revealed that migraine significantly reduced the risk of dementia and Alzheimer's disease in individuals without the *APOE*‐ε4 allele, but the association reversed in those with one or two ε4 alleles, though the confidence intervals were large and non‐significant; migraine also reduced AD risk in those without diabetes, but the effect was non‐significant in those with diabetes (Table S1). Moreover, adjusting for covariates excluded from the main analyses did not change the results, which remained significant (Table S2). Lastly, in a subgroup analysis of migraine patients (*N* = 1041, 47 dementia cases), we found that migraine medication use was slightly associated with a higher risk of dementia (HR 1.13, CI: 0.62‐2.04) but the association was not statistically significant, likely due to the low number of events.

## 4 DISCUSSION

In this study, we found that individuals with migraine have a reduced risk of developing dementia and AD, compared to people without migraine.

Our results differ from some findings reported in the literature, which suggest an increased risk of dementia for people with migraine.^5^ Several factors may contribute to this discrepancy. Firstly, literature shows considerable heterogeneity,^3^ with case –control and retrospective cohort studies suggesting an association between migraine and dementia. However, results from these studies have erroneously been given a causal interpretation of an increased risk. A critical evaluation of the results from one of the latest meta‐analysis suggest that, in prospective studies, the risk of dementia in people with migraine seems to be decreased rather than increased.^3^ Our findings provide further evidence that the true association between migraine and dementia may indeed be a reduced risk. This discrepancy between the different study designs may be the result of recall bias in retrospective designs.^36^ Secondly, some studies lack a follow‐up duration that also encompasses prodromal dementia, to explore the temporal relationship between migraine and dementia.^5^ Lastly, selection bias in clinic‐based studies may influence the selection of patients into these studies. Overall, our results are in line with the findings from prospective studies, that participants with migraine may have a reduced risk of dementia and AD.

Whether this reduced risk is due to a true biological mechanism or due to other methodological issues is not yet clear and needs further investigation. However, this lack of a positive association is in line with previous findings from the Rotterdam Study, where, compared to controls, participants with migraine have higher cerebral blood flow,^37^ better cognition (better performance on tests of executive function and fine motor skills),^38^ and less calcification in internal carotid arteries.^39^ In addition, no statistically significant difference has been found between participants with and without migraine, in terms of cerebral small vessel disease,^40^ retinopathy^41^ and stroke risk.^9^ These findings, combined with the results of the present study, necessitate the consideration of the possibility that migraine may have a protective effect against dementia. It has been suggested that the migraine attack serves as a neuroprotective response to oxidative stress in the brain.^42^ Evidence from different lines of research indicates that the components of migraine pathophysiology may have the net effect of reducing the oxidative stress in the brain.^42^ This is relevant because recent reviews underline the crucial role of oxidative stress in dementia and AD.^43^, ^44^ We may speculate that, similar to other defensive mechanisms (e.g., fever or swelling), migraine attacks may be protective at moderate levels but damaging when excessively intense.^42^ Nevertheless, this observation needs further confirmation in other population‐based studies.

This study has a number of strengths. Firstly, we used a validated questionnaire for migraine assessment thereby reducing the possibility of (non‐differential) misclassification bias. Secondly, our recruitment of participants from the general population, without any predetermined exclusion criteria, enabled us to capture migraine patients who have not yet visited a healthcare professional, which is the case in more than half of migraine sufferers according to large‐scale surveys.^45^, ^46^ This reduces the risk of selection bias. Lastly, dementia cases were established using an adjudication method which includes consensus meetings and, when necessary, neuroimaging‐aided diagnosis.

This study also has several limitations that should be taken into consideration. First, while our recruitment from the general population is a strength in reducing selection bias, it may introduce heterogeneity in the measure of migraine exposure. We used a slightly modified version of the ICHD‐2 criteria where only participants who reported “severely” painful headaches in a screening question were further assessed, which likely excluded milder cases typically captured under standard “moderate‐to‐severe” criteria. While this limits the applicability of our findings to those with moderate or mild migraine, our questionnaire likely captured subset of people with migraine who are missed by hospital based studies but are also have more than moderate headache severity. This is an important distinction, as some studies suggest that severe migraine (measured by number of hospital‐visits), may be linked to a higher dementia risk.^47^ Therefore, any misclassification bias resulting from our exclusion of milder cases would likely make our results appear weaker rather than stronger. Nonetheless, future studies should collect detailed data on attack frequency, severity, and chronicity to better characterize this exposure.

Second, although our data in this study are from a prospective cohort study, there might be a subset of participants for whom migraine status would have been assessed retrospectively as our questionnaire focused on lifetime symptoms. At the time of assessment (between 2006 and 2011) participants from the original and second cohort would have aged into their 70s, and 60s, respectively. At these ages, subclinical manifestations of dementia may begin affecting participants’ recollection and, as such, misclassify their exposure status. However, previous findings from the Rotterdam study suggest that migraine is associated with better cognitive performance, as evidenced by higher mini‐mental state examination scores and global cognition in participants with migraine.^38^ This suggests that reverse causation is unlikely to explain our findings. Additionally, we conducted cohort and age stratified analyses, they show no statistically significant differences among the groups.

Third, it may be argued that the use of migraine medication may explain the reported protective effect rather than the migraine itself. We lacked detailed information on specific migraine drugs and dosages, as only a subset of participants reported medication use. Subgroup analysis of this subset of participants, showed that while migraine medication use was slightly associated with a higher dementia risk, the association was not statistically significant. Moreover, a recent review^48^ suggests that, the most commonly used migraine medications, triptans and ergotamines may have detrimental cognitive effects, while gepants appear neutral and non‐steroidal anti‐inflammatory drugs (NSAIDs) show inconsistent results. Given these findings, migraine medications are unlikely to explain the reduced dementia risk observed in our cohort, though further studies should examine their role in more detail.

Fourth, it would be interesting to study the differences among migraine subgroups (probable migraine, migraine with aura, active migraine) in terms of dementia risk. While we identified these subgroups, the relatively short follow‐up duration limited the number of dementia events, which precluded meaningful stratified analyses. A longer follow‐up period could have provided additional events, thereby enabling more detailed stratification by migraine subtypes. However, the study's timeline and the finalization of the dataset in 2020 restricted the possibility of further extending the follow‐up. Additionally, although vascular dementia has been associated with migraine in previous studies, only one participant with migraine developed vascular dementia during the follow‐up, which was insufficient for meaningful analysis in this cohort. The heterogeneity of results in the literature regarding the risk of vascular dementia in migraine further complicates this analysis, as various studies have reported inconsistent findings on this association.^1^ Thus, further studies with larger sample sizes and longer follow‐up periods are needed to explore the relationship between migraine and dementia subtypes, including vascular dementia.

Finally, despite the statistical adjustment for possible confounders, there might be a risk of residual confounding. Yet, we believe this is not likely since the unmeasured factors would be very strongly associated with both migraine and dementia to explain our results.

In conclusion, our study indicates that individuals with migraine may have a reduced risk of developing dementia including its major subtype Alzheimer's disease.

## CONFLICT OF INTEREST STATEMENT

The authors declare that there is no conflict of interest. Author disclosures are available in the Supporting information.

## CONSENT STATEMENT

The Rotterdam Study has been approved by the Medical Ethics Committee of the Erasmus MC (registration number MEC 02.1015) and by the Dutch Ministry of Health, Welfare and Sport (Population Screening Act WBO, license number 1071272‐159521PG). The Rotterdam Study Personal Registration Data collection is filed with the Erasmus MC Data Protection Officer under registration number EMC1712001. The Rotterdam Study has been entered into the Netherlands National Trial Register (NTR; www.trialregister.nl) and into the WHO International Clinical Trials Registry Platform (ICTRP; https://apps.who.int/trialsearch/) under shared catalogue number NTR6831. All participants provided written informed consent to participate in the study and to have their information obtained from treating physicians.

## Supporting information



Supporting Information

Supporting Information
