# Supplementary material for: Migraine and the Risk of Dementia in the General Population
Source: Alzheimers Dement. 2026 Apr 23;22(4):e71386. doi: 10.1002/alz.71386 (PMC13106216; doi:10.1002/alz.71386)
Supplement: Supplementary file 1 — Supporting Information [file ALZ-22-e71386-s002.docx]

**Table S1.** Cox regression analyses for the association of migraine with the risk of dementia and Alzheimer’s disease, stratified by potential effect modifiers.

| Migraine | Hazard ratio (95 % Confidence Interval) | | | |
| --- | --- | --- | --- | --- |
|  | Cases/N | Dementia | Cases/N | Alzheimer’s Disease |
| *APOE-ε4* carriership^a^ |  |  |  |  |
| No *ε4* allele | 287/4959 | **0.59 (0.39 – 0.91)** | 208/4959 | **0.49 (0.28 – 0.84)** |
| One *ε4* allele | 179/1774 | 1.43 (0.77 – 2.64) | 149/1774 | 1.42 (0.67 – 3.01) |
| Two *ε4* allele | 25/155 | 1.03 (0.13 – 8.01) | 22/155 | 1.41 (0.18 – 11.38) |
| Hypertension^b^ |  |  |  |  |
| No | 66/2172 | 0.69 (0.30 – 1.61) | 50/2172 | 0.48 (0.15 – 1.56) |
| Yes | 425/4716 | 1.01 (0.41 – 2.50) | 329/4716 | 1.23 (0.36 – 4.22) |
| Diabetes Mellitus^b^ |  |  |  |  |
| No | 376/5924 | 0.73 (0.52 – 1.02) | 292/5924 | **0.57 (0.38 – 0.87)** |
| Yes | 115/964 | 0.87 (0.38 – 2.01) | 87/964 | 1.17 (0.46 – 2.97) |
| Smoking ^c^ |  |  |  |  |
| Never | 167/2285 | 0.72 (0.43 – 1.22) | 129/2285 | 0.54 (0.27 – 1.06) |
| Past | 266/3452 | 0.91 (0.47 – 1.75) | 208/3452 | 1.17 (0.51 – 2.66) |
| Current | 58/1151 | 1.13 (0.39 – 3.23) | 42/1151 | 0.80 (0.17 – 3.89) |
| Study Cohort^d^ |  |  |  |  |
| First | 278/1509 | 0.71 (0.48 – 1.07) | 219/1509 | 0.67 (0.42 – 1.05) |
| Second | 148/1802 | 0.82 (0.40 – 1.68) | 114/1802 | 0.47 (0.17 – 1.29) |
| Third | 65/3577 | 1.20 (0.52 – 2.79) | 46/3577 | 1.19 (0.42 – 3.36) |
| Age range^e^ |  |  |  |  |
| 45-64 | 23/3268 | 1.11 (0.38 – 3.26) | 13/3268 | 1.54 (0.42 – 5.61) |
| 65-84 | 371/3234 | 0.54 (0.17 – 1.69) | 289/3234 | 0.30 (0.08 – 1.19) |
| 85+ | 97/386 | 0.58 (0.16 – 2.09) | 77/386 | 0.38 (0.08 – 1.71) |

**^a^** Models were adjusted for age, sex, educational level, body mass index smoking status.
**^b^** Models were adjusted for age, sex, educational level, smoking status, body mass index and *APOEε4* carriership.
**^c^** Models were adjusted for age, sex, educational level, body mass index and *APOEε4* carriership.
**^d^** Models were adjusted for age, sex, educational level, smoking status, body mass index and *APOEε4* carriership.
**^e^** Models were adjusted for sex, educational level, smoking status, body mass index and *APOEε4* carriership.

**Table S2.** Cox regression analyses for the association of migraine and the risk of all-cause and Alzheimer’s Disease dementia.

|  | Hazard ratio (95 % Confidence Interval) | |
| --- | --- | --- |
| Migraine | All-cause Dementia (cases/N: 491/6888) | Alzheimer’s Disease  (cases/N: 379/6888) |
| Model 1 (crude) | 0.58 (0.43 – 0.78) | 0.49 (0.34 – 0.70) |
| Model 2^a^ | 0.71 (0.52 – 0.96) | 0.60 (0.41 – 0.86) |
| Model 3^b^ | 0.70 (0.51 – 0.95) | 0.58 (0.40 – 0.85) |
| Model 4^c^ | 0.71 (0.52 – 0.97) | 0.59 (0.41 – 0.86) |

**^a^**Model 2: adjusted for age and sex.
^b^Model 3: Model 1 + educational level, smoking status, body mass index and APOEε4 carriership adjusted.
**^c^Model 4:** Model 3+ coronary heart disease history, hypertension, hypercholesterolemia, diabetes mellitus, alcohol use, marital status, depressive symptoms and anxiety disorder diagnosis adjusted.
